# Cysteinyl leukotrienes: multi-functional mediators in allergic rhinitis

**DOI:** 10.1111/j.1365-2222.2006.02498.x

**Published:** 2006-06-01

**Authors:** M Peters-Golden, MM Gleason, A Togias

**Affiliations:** * University of Michigan Ann Arbor, MI, USA; † Merck & Co., Inc. West Point, PA, USA; ‡ Johns Hopkins University Baltimore, MD, USA

**Keywords:** allergic rhinitis, cysteinyl leukotrienes, CysLT_1_ receptor, eosinophils, inflammation, leukotriene C4 synthase, 5-lipoxygenase

## Abstract

Cysteinyl leukotrienes (CysLTs) are a family of inflammatory lipid mediators synthesized from arachidonic acid by a variety of cells, including mast cells, eosinophils, basophils, and macrophages. This article reviews the data for the role of CysLTs as multi-functional mediators in allergic rhinitis (AR). We review the evidence that: (1) CysLTs are released from inflammatory cells that participate in AR, (2) receptors for CysLTs are located in nasal tissue, (3) CysLTs are increased in patients with AR and are released following allergen exposure, (4) administration of CysLTs reproduces the symptoms of AR, (5) CysLTs play roles in the maturation, as well as tissue recruitment, of inflammatory cells, and (6) a complex inter-regulation between CysLTs and a variety of other inflammatory mediators exists.

## Introduction

Allergic rhinitis (AR), which affects approximately 20% of the population in industrialized countries, is associated with substantial morbidity, primarily in the context of reduced quality of life and productivity. Patients with AR experience increased incidence of acute sinusitis and otitis media, both of which can be regarded as causatively linked to nasal disease. In addition, AR is closely related to asthma: more than 80% of patients with atopy and asthma have some form of nasal disease, and the prevalence of asthma in patients with AR can reach 40%, at least fivefold greater than that observed in the general population [1, 2]. Rhinitis also is a major risk factor for the development of asthma. Finally, AR is a prototype of immediate hypersensitivity, and understanding its pathophysiology is of significance for the entire spectrum of allergic conditions.

Identified in the late 1970s [3], leukotrienes are a family of inflammatory lipid mediators synthesized from arachidonic acid by a variety of cells, including mast cells, eosinophils, neutrophils, basophils, and macrophages. The cleavage of arachidonic acid from the nuclear membrane by phospholipase A_2_ (PLA_2_) initiates the synthesis of the leukotrienes [4]. The subsequent interaction of arachidonic acid with the biosynthetic proteins 5-lipoxygenase (5-LO) and 5-lipoxygenase activating protein (FLAP) forms the intermediate 5-HPETE (5-hydroxyperoxy-6,8,11,14-eicosatetraenoic acid), which is quickly converted to LTA_4_. LTA_4_ can be converted to LTB_4_ by LTA_4_ hydrolase or to LTC_4_ by LTC_4_ synthase. LTC_4_ is converted extracellularly to LTD_4_ and LTE_4_ by sequential amino acid removal from the glutathione tripeptide moiety. LTC_4_ is converted to LTD_4_ through removal of glutamic acid by γ-glutamyl transpeptidase. Glycine is then removed from LTD_4_ by dipeptidase. Consequently, LTC_4_, LTD_4_, and LTE_4_ are together referred to as cysteinyl leukotrienes (CysLTs). LTE_4_ is the most stable of the CysLTs and can be measured after excretion into the urine; urinary LTE_4_ is often used as a marker of ‘whole body’ leukotriene synthesis. LTB_4_ contains no cysteine, and is, therefore, not a CysLT.

CysLTs exert their actions through activation of two G-protein-coupled receptors: CysLT subtype 1 receptor (CysLT_1_) and CysLT_2_. CysLT_1_ is the most studied and is the target for the drugs montelukast, zafirlukast, and pranlukast. As such, its role in AR and other conditions is better understood. By contrast, there is a paucity of information about the role of CysLT_2_, in part because no specific antagonists for this receptor are yet available. Both receptors are present in inflammatory cells, blood vessels, and nasal glandular cells [5, 10]. CysLT_1_ binds LTD_4_ with much greater affinity than either LTC_4_ or LTE_4_ [11]; in contrast, CysLT_2_ binds LTC_4_=LTD_4_>LTE_4_. Signaling through both subtypes of the CysLT receptor is mediated, in part, through intracellular calcium mobilization.

CysLTs were originally established as mediators of asthma. However, AR involves immunologically similar reactions, and it was only logical to assume that the CysLTs would be important mediators in this condition. CysLTs and leukotriene receptor occupancy have been linked to several processes in AR, including: (1) dilation of nasal blood vessels and vascular permeability with oedema formation, both leading to nasal congestion, (2) increased mucus production and secretion, leading to rhinorrhea, and (3) recruitment of inflammatory cells from the bloodstream into tissue, thus perpetuating the inflammatory response. However, there is a growing body of evidence suggesting that CysLTs are multi-functional mediators playing a broader role in the inflammation that characterizes allergic disorders such as AR.

This article reviews the data for the role of CysLTs as multi-functional mediators in AR. We will review the evidence that: (1) CysLTs are released from inflammatory cells that participate in AR, (2) receptors for CysLTs are located in nasal tissue, (3) CysLTs are increased in patients with AR and are released following allergen exposure, (4) CysLTs produce symptoms of AR, (5) CysLTs play a role in bone marrow production and tissue recruitment of inflammatory cells, and (6) there is a complex inter-regulation between CysLTs and a variety of other inflammatory mediators.

## Cells that are linked to the pathogenesis of allergic rhinitis produce and release cysteinyl leukotrienes

Mast cells, basophils, eosinophils, dendritic cells, monocytes/macrophages, and T lymphocytes collectively initiate and perpetuate mucosal inflammation in AR. The IgE-bearing mast cells and basophils have the greatest capacity to produce CysLTs, but eosinophils, dendritic cells, monocytes/macrophages, and T lymphocytes also have been shown to release CysLTs (Table 1). Basophils produce more than 100-fold higher amounts of CysLTs compared with eosinophils [12, 13]. Eosinophils isolated from patients with AR released significantly higher levels of CysLTs than eosinophils isolated from healthy subjects following stimulation with the calcium ionophore A23187 [14]. Recently, expression of the CysLT biosynthetic proteins 5-LO, FLAP, and LTC_4_ synthase was demonstrated in inflammatory cells present in the nasal secretions of symptomatic patients with seasonal AR [15]. Most of the cells expressing these proteins were eosinophils and mononuclear cells; interestingly, only 30% of mast cells and basophils expressed these enzymes. Many of the same inflammatory cells that secrete CysLTs also express the cell surface CysLT_1_ receptor (Table 1), suggesting an autoregulatory mechanism.

**Table 1:** Studies demonstrating cells that express the CysLT_1_ receptor and cells that synthesize cysteinyl leukotrienes (CysLTs)

		CysLT systhesis	
		
Cell type	Express CysLT_1_receptor	Production of CysLTs	Presence of CysLT synthetic enzymes
Basophils	[15, 17]	[176–178]	5-LO, FLAP, LTC4 Syn [15]
Mast Cells	[15, 16, 18, 20, 179]	[57, 129, 176, 180, 181, 182]	5-LO, FLAP, LTC4 Syn [15, 57]
Monocytes	[5, 15, 16, 17]	[183, 184]	5-LO, FLAP, LTC4 Syn [15]
Eosinophils	[5, 15, 16, 17,20, 119, 156, 185]	[14, 163, 186, 187, 188, 189, 190]	5-LO, FLAP, LTC4 Syn [15]
Dendritic cells	[39, 40]	[39]	5-LO and FLAP [42, 191]; FLAP, 5-LO and LTC4 Syn [39]
Macrophages	[11, 15, 16, 17,20, 21, 192]		5-LO, FLAP, LTC4 Syn [15]
T lymphocytes	[5, 20, 193]	[194]	
B lymphocytes	[17]		
Neutrophils	[5, 15, 16, 20]	[195]	5-LO, FLAP [15]
Haematopoietic stem cells	[17, 19]	[91, 93]	5-LO [19]
Epithelial cells	—	[196]	LTC4 Syn [15]
Glandular epithelium	[16, 69]		
Endothelial cells	[16, 197]	[91, 197]	
Smooth muscle cells	[11, 17]	—	

CysLT_1_ receptor, cysteinyl leukotriene subtype 1 receptor; 5-LO, 5-lipoxygenase enzyme; FLAP, 5-lipoxygenase activating protein; LTC_4_Syn, LTC_4_synthase.

## Receptors for cysteinyl leukotrienes are found in tissue and on cells that are involved in allergic rhinitis inflammation and symptoms

Using *in situ* hybridization and immunohistochemical techniques, the CysLT_1_ receptor has been localized to nasal mucosal interstitial cells, glandular epithelium, and a variety of inflammatory cells (Table 1). Mast cells, neutrophils, eosinophils, monocytes, and macrophages isolated from nasal lavage fluid of patients with active AR express the CysLT_1_ receptor [15]. CysLT_1_ receptor mRNA and protein have been found on blood vessels, interstitial cells, eosinophils, mast cells, monocytes/macrophages, neutrophils, and glandular and vascular endothelium of human nasal mucosal tissue of patients undergoing turbinectomy [16]. Using a panel of peripheral blood cell markers, the presence of the CysLT_1_ receptor also has been demonstrated on circulating eosinophils, B lymphocytes, basophils, monocytes, macrophages, and on CD34^+^ haematopoietic stem cells [5, 15, 17–20].

CysLT_1_ expression is subject to regulation *in vitro* and *in vivo*. For example, cytokines have been shown to enhance CysLT_1_ expression in leucocytes and mesenchymal cells *in vitro* [21]. Sousa et al. [20] studied the expression and regulation of the CysLT_1_ receptor on nasal mucosal inflammatory cells from aspirin-sensitive and non-aspirin-sensitive patients with rhinosinusitis and polyps treated with lysine aspirin or placebo. Compared with the non-aspirin-sensitive patients, the absolute number of cells and the percentage of CD45^+^ leucocytes expressing the CysLT_1_ receptor, but not the LTB_4_ receptor, was higher in the aspirin-sensitive patients. Desensitization with lysine aspirin selectively reduced the number of CD45^+^ leucocytes expressing the CysLT_1_ receptor, but not the LTB_4_ receptor, suggesting a specific receptor-regulating mechanism associated with the therapeutic benefit of aspirin desensitization in patients with asthma and AR [22]. These data by Sousa and coworkers are the first to demonstrate that CysLT_1_ expression can be modulated in disease states and suggest that down-regulation of CysLT_1_ receptor could represent a mechanism for therapeutic benefit (in this case, by aspirin desensitization).

CysLT_2_ receptors are broadly distributed not only in leucocytes, but also in heart tissue, brain, adrenal glands, and vasculature. Recent studies in mice with deletion [23] or overexpression [24] of CysLT_2_ suggest a prominent role for this receptor in mediating vascular permeability, a process to which CysLT_1_ also clearly contributes [25] Emerging data suggest that CysLT_2_ may also contribute to fibroproliferation [23, 26] and to inflammatory responses [6] in a manner distinct from CysLT_1_.

## Cysteinyl leukotrienes are found in patients with allergic rhinitis

Several studies have demonstrated that CysLT levels in nasal fluids are increased in patients with AR (Table 2). CysLTs are significantly elevated in nasal lavage fluid from symptomatic allergic rhinitic patients compared with that from healthy controls [27–29], as well as in nasal lavage fluids during the early and late allergic responses [30–33]. CysLTs were elevated in nasal secretions within 5 min [33] and persisted for 30 min [31] following allergen exposure, and these levels correlated with the duration of symptoms [31]. Ragweed challenge elevated CysLT concentrations in a dose-dependent manner in patients with AR [30, 31], whereas challenge with methacholine [34] or non-relevant allergen [35] had no effect. CysLT levels fluctuated with seasonal allergen exposure [33, 36] and correlated with symptom scores in individuals with AR, but not in non-allergic controls [37]. Levels of CysLTs were also found to increase in nasal fluids when reactions to cold, dry air take place, presumably as a result of mast cell degranulation [38]. This raises the possibility that CysLTs may participate in some forms of rhinitis in the absence of allergic reactions.

**Table 2:** Cysteinyl leukotrienes (CysLTs) are elevated in patients with allergic rhinitis and conjunctivitis

CysLTs are elevated in	Studies
Nose during natural/seasonal allergen exposure	[27–29,^36^, ^37^, ^46^, ^116^, ^198^]
Urine during natural/seasonal allergen exposure	[72]
Nose after allergen challenge	[30, 31, 32, 33, 34, 35,199, 200, 201]
Eyes after allergen challenge	[202, 203]

## Cysteinyl leukotrienes may be participating in the process of allergic sensitization

An allergic response requires processing of the allergen by an antigen-presenting cell. Dendritic cells are potent antigen-presenting cells, initiating the immune response by taking up and presenting antigen to and influencing the polarization of T cells. The effect of CysLTs on dendritic cell function has recently been explored. Dendritic cells express the CysLT_1_ receptor [39–41] and the enzymatic machinery necessary to produce CysLTs [39, 41, 42]. CysLTs have been shown to modulate allergen-stimulated dendritic cell production of interleukin (IL) 10, IL-12, IL-5, and interferon γ (IFN-γ) [39] and to enhance dendritic cell-stimulated antigen presentation, T cell proliferation, and T cell cytokine production [41, 43, 44]. They also directly promote dendritic cell migration [40, 45]. CysLTs may influence dendritic cell migration indirectly by increasing the production of dendritic cell chemoattractants, including RANTES [46, 47], macrophage-inflammatory-protein (MIP)-1α [40, 48], and MIP-3α [40] from monocytes and macrophages. However, in a recent study, CysLT_1_ receptor antagonists did not affect cytokine production by monocyte-derived dendritic cells or monocyte-derived dendritic cell effects on CD4^+^ lymphocytes [41].

## Cysteinyl leukotrienes can produce symptoms of allergic rhinitis

Experimental exposure of the nasal mucosa to allergens in sensitized individuals with AR initiates a dual-phase immune response [49]. The early or immediate phase response occurs within minutes of allergen exposure and is characterized primarily by sneezing, nasal pruritus, rhinorrhea, and acute congestion. The late-phase response occurs hours after allergen exposure and is mainly associated with congestion and, to a lesser extent, rhinorrhea and sneezing.

Upon allergen exposure, crosslinking of IgE receptor activates mast cells and initiates the early allergic response through immediate release of preformed mediators, including histamine, proteases (e.g., tryptase), and tumour necrosis factor α (TNF-α), and the release of newly synthesized mediators, including CysLTs and prostaglandin D_2_. CysLTs are released from mast cells within minutes of allergen exposure (Table 3).

**Table 3:** Allergen-induced rhinitis and clinical rhinitis outcomes affected by cysteinyl leukotrienes (CysLTs)

Symptom	Studies showing effect
Sneezing	Significantly correlated with CysLTs levels in patients with allergic rhinitis following allergen challenge [30]
	Significantly improved with LTRA in clinical studies of patients with allergic rhinitis [46, 52–56, 61]
Rhinorrhea	Significantly worsened with intranasal CysLT application [31, 50]
	Significantly improved with LTRA in studies of patients with allergic rhinitis following allergen challenge [67]
	Significantly improved with LTRA in clinical studies of patients with allergic rhinitis [46,[52–56, 6161,^68^68]
Nasal pruritus	Significantly improved with LTRA in clinical studies of patients with allergic rhinitis [53, 54, 61, 62]
Congestion	Significantly worsened with intranasal CysLT application [31, 50, 51, 204, 205]
	Significantly improved with LTRA in clinical studies of patients with allergic rhinitis [46, 52–56, 61, 67, 68, 74]
Itchy throat and palate	Significantly improved with LTRA in clinical studies of patients with allergic rhinitis [52]
Eye symptoms	Significantly improved with LTRA in clinical studies of patients with allergic rhinitis [53–56, 61, 143, 206]
Rhinoconjunctivitis quality of life	Significantly improved with LTRA in clinical studies of patients with allergic rhinitis [53–56, 61, 206]

LTRA, leukotriene receptor antagonist.

Although sneezing occurs within 1–2 min of allergen exposure and decreases rapidly thereafter, some sneezing can occur during the late-phase response. After allergen challenge, the timing of LTC_4_ release has been shown to correlate with sneezing [30, 33]. CysLTs do not directly induce sneezing and pruritus [50, 51]; however, CysLTs may have an indirect effect on sneezing, as indicated by the reduction of sneezing with zafirlukast [52] and montelukast [46, 53, 54, 55, 56, 57], both leukotriene receptor antagonists, in clinical trials of patients with AR.

Nasal pruritus occurs exclusively during the early-phase response as nerve fibres, probably stimulated by histamine, elicit this sensation. The role of leukotrienes in nasal pruritus is not defined. However, the ability of leukotriene receptor antagonists to relieve the itch of atopic dermatitis [58] and chronic idiopathic urticaria [59, 60] suggests that leukotrienes may contribute to nasal pruritus. This hypothesis is further supported by the ability of montelukast to reduce nasal pruritus in clinical trials of patients with seasonal AR [53, 54, 61, 62].

CysLTs do not directly stimulate sensory nerves. However, in the presence of CysLTs, an electrical stimulus releases increased amounts of neuropeptides from tachykinergic nerves [63, 64]. This suggests that CysLTs may potentiate neural phenomena such as neurogenic inflammation, which appear to be increased in individuals with AR [65, 66]. In addition, the *in vivo* responsiveness of nasal sensory nerves to histamine may become increased in the presence of CysLTs, as suggested by the work of Konno et al. [67].

Rhinorrhea, resulting from increased glandular activity, is predominantly an early-phase symptom, but it can also occur during the late phase. Application of LTD_4_ to the nasal mucosa of patients with AR increased the amount of nasal secretions in a dose-dependent manner, an effect that peaked within 5 min of mediator application [31, 50]. The reduction in rhinorrhea with pranlukast [67], zafirlukast [52], and montelukast [46, 53, 54, 55, 56,61, 68] in clinical trials of patients with AR further supports a role for CysLTs in stimulating nasal secretions. This effect is probably direct, given the fact that the CysLT_1_ receptor has been found on human nasal mucosal glands [16, 69].

Nasal congestion is prominent during both the early- and the late-phase response to allergen. The late-phase response occurs in approximately 50% of allergic patients [70]. CysLTs have been shown to cause prolonged congestion (Table 3). CysLTs also increase vascular permeability [71], and the resulting oedema may contribute to the narrowing of nasal passages. Five minutes after topical application of LTD_4_, nasal mucosal blood flow and nasal airway resistance increased in a dose-dependent manner [31, 51]. In the study by Okuda et al. [50], the increase in nasal airway resistance did not abate for several hours. Histamine also increases nasal airway resistance, albeit to a maximum at 20 min after application [31]. Urinary LTE_4_ levels were found to be significantly higher in patients with AR with severe nasal congestion [72] and less evident in patients with mild congestion [73]. The improvement in nasal congestion following treatment with leukotriene modifiers, measured either by symptom scores [46, 52, 53, 54, 55, ] or airway resistance [61, 67, 68] in clinical trials of patients with AR further implicates CysLTs in mediating nasal congestion. It should be noted that, because of the presence of both CysLT_1_ and CysLT_2_ receptors in nasal vasculature, and because stimulation of the CysLT_2_ receptor appears to increase vascular permeability [24], antagonism of both receptors may offer stronger effects against nasal congestion in AR.

In support of the contribution of CysLTs in mediating individual symptoms of the early- and late-phase allergic response, several CysLT_1_ receptor antagonists have been shown to reduce the aggregate of symptoms in clinical trials of patients with AR (Table 3). Pranlukast improved daytime symptoms [75], and zafirlukast improved nasal congestion, sneezing, rhinorrhea, and itchy nose, throat, and palate, although no clear dose-response could be generated [52]. Montelukast has been shown to improve daytime symptoms (congestion, rhinorrhea, sneezing, and nasal pruritus), night-time symptoms (difficulty to sleep, awakenings, and congestion upon awakening), daytime eye symptoms (tearing, itchy, red, and puffy eyes), and quality of life [53–56].

## Cysteinyl leukotrienes and cellular inflammation in allergic rhinitis

**Fig. 1:**
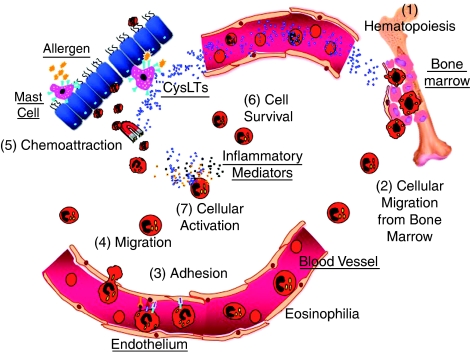
Cysteinyl leukotrienes (CysLTs) and the Inflammatory Events of Allergic Rhinitis. Crosslinking of immunoglobulin E with allergen initiates release of a variety of mediators from mast cells, including CysLTs. CysLTs play a role in hematopoiesis, cellular migration from bone marrow to the circulation, adhesion of inflammatory cells to the vascular endothelium, migration of cells to the nasal tissue, cell survival, and cellular activity enhancement.

In the course of natural exposure to aeroallergens, as well as with experimental allergen challenge, various inflammatory cells, including eosinophils, basophils, monocytes, and TH_2_ lymphocytes, are elevated in nasal tissue and nasal secretions [76, 77] and correlate with symptoms in patients with AR [78, 81]. Inflammatory cells release various forms of mediators into the nasal mucosa, ranging from symptom-producing substances to pure cytokines that perpetuate chronic inflammation and symptoms. The steps leading to inflammatory cell recruitment are not completely understood, and it is quite likely that the mechanisms of recruitment and activation are unique for each cell type. There is enough evidence in both asthma and AR to support the hypothesis that inflammatory elements generated during local allergic reactions may produce systemic signals affecting circulating cells, cells residing in peripheral lymphoid tissue, and immature cells residing in the bone marrow [2, 9, 82, 83, 84]. When contemplating the continuously emerging knowledge on the immunomodulatory properties of the CysLTs, it is reasonable to put forward a hypothesis that these mediators contribute to the systemic inflammation associated with AR. This hypothesis is schematically depicted in Fig. 1.

### Step 1: haematopoiesis

The role of eosinophil and basophil progenitors in allergic inflammation and their fluctuation with seasonal exposure has been reviewed [85–87]. CysLTs have been shown to play a role in leucopoiesis induced by granulocyte-macrophage colony stimulating factor (GM-CSF) [88–90], IL-5 [89], and IL-3. [91] In a mouse model of AR, montelukast was shown to inhibit either bone marrow IL-5- or GM-CSF-responsive eosinophil/basophil colony-forming units and IL-5-stimulated eosinophil maturation [92]. The inhibition of IL-5-dependent proliferation of bone marrow eosinophil–basophil progenitors and GM-CSF-dependent proliferation of peripheral blood eosinophil–basophil progenitors by the leukotriene receptor antagonist montelukast [89] points to the activity of CysLTs through the CysLT_1_ receptor on CD34^+^ haematopoietic bone marrow stem cells [17, 19, 93]. Interestingly, these cells express 5-LO [19, 94, 95], and bone marrow cells can produce CysLTs upon *in vitro* stimulation with the calcium ionophore A23187 [91, 93]. These data suggest that CysLTs may be both paracrine and autocrine contributors to haematopoiesis.

### Step 2: migration from bone marrow

Chemotaxis and transendothelial migration of CD34^+^ progenitor cells in response to LTD_4_ and inhibition by the leukotriene receptor antagonist MK-571 [19] suggest a role for CysLTs in leucocyte migration from the bone marrow into the circulatory system. Chemotaxis and transendothelial migration are preceded by endothelial adhesion. LTD_4_ up-regulated adhesion of human peripheral blood CD34^+^ progenitors to bone marrow endothelium; this was blocked by MK-571 and antibodies against β_1_ and β_2_ integrins [96].

### Step 3: adhesion to post-capillary venules

Leucocyte adhesion to the vascular wall is the first step in recruitment and migration into nasal tissue. Adhesion molecules are expressed by the nasal endothelium of patients with AR within 24 h after nasal allergen challenge [97]. CysLTs enhance leucocyte adhesion by increasing the expression of the adhesion molecules P-selectin and soluble sialyl Lewis^x^ [98, 99], αMβ_2_ [100], β_2_ integrins [101], and Mac-1 [102]. CysLT-induced leucocyte adhesion and adhesion molecule expression is inhibited by the leukotriene receptor antagonists montelukast [102] and pranlukast [101]. Nagata et al. [103] observed that eosinophil adhesion via β_2_ integrins to intercellular cell adhesion molecule 1 (ICAM-1) augmented eosinophil LTC_4_ generation. These data suggest a positive feedback mechanism that increases the production of CysLTs at the site of eosinophil adhesion.

### Steps 4 and 5: migration and chemoattraction

Transendothelial migration of leucocytes across the vessel wall into the tissue follows cellular adhesion. CysLTs are direct chemoattractants for eosinophils and have been shown to enhance eosinophil migration *in vivo* [104–106] and *in vitro* [92, 102, 106, 107]. This phenomenon is dose-dependently inhibited by leukotriene receptor antagonism with FPL 55712 [106], SK&F 104353 [107] and montelukast [102, 108]. Eotaxin is a selective chemoattractant for eosinophils. The role of CysLTs in eosinophil recruitment is further implicated by the observation that LTC_4_ increases eotaxin release from endothelial cells [109, 110] and from IL-13-primed fibroblasts [111], which is blocked by montelukast and pranlukast. Finally, montelukast treatment has been shown to reduce eosinophils in nasal mucosa of adults [46] and children [61] with AR.

### Step 6: cell survival

Tissue eosinophilia is a function of both the influx of eosinophils into the nasal mucosa as well as their half-life (survival). CysLTs increase eosinophil survival time [112], and this effect is inhibited by leukotriene receptor antagonists [112, 113].

### Step 7: cellular activation

Once in the nasal tissue, CysLTs also promote inflammation by enhancing the activity of inflammatory cells. This section focuses on eosinophil activation, but the ability of CysLTs to affect the function of other inflammatory cells, including monocytes, basophils, mast cells, and T lymphocytes, is also described.

Activated eosinophils release a variety of inflammatory mediators and probably play a significant role in allergic disease. For example, eosinophilic cationic protein (ECP) is toxic to epithelial tissue; a consequence of such toxicity may be exposure of sensory nerve fibres to environmental irritants. Major basic protein (MBP), on the other hand, can inhibit the ability of acetylcholine to prevent further acetylcholine release from peripheral parasympathetic nerves by deactivating the M2 receptor [114]. Elevated ECP in the nasal fluid of patients with AR [115] correlates with an increase in LTC_4_ [116], and treatment with montelukast decreases ECP levels in the serum of adults [117] and in nasal washes from pediatric patients [118]. A significant correlation between CysLTs and eosinophilic protein X, a marker of eosinophilic activity, has also been demonstrated [27]. Superoxide radicals mediate inflammation through oxidative damage in cells, and LTD_4_ was shown to increase superoxide radical levels in eosinophils *in vitro* [100]. Eosinophil-derived neurotoxin (EDN) is another cytotoxic mediator. IL-5-induced release of EDN was enhanced by LTD_4_ [119] and, in another study, LTD_4_-induced EDN release by peripheral blood eosinophils of healthy subjects [120]. The effects of LTD_4_ on superoxide radicals and EDN were blocked by pranlukast [120].

In clinical studies, the leukotriene receptor antagonist montelukast reduced peripheral blood eosinophil numbers in adults [53–56] and children [61, 121] with AR. Taken together, the effects of CysLTs on eosinophil differentiation, maturation, proliferation, adhesion molecule expression, migration, survival, and activation described above are consistent with a role of these mediators in local and systemic allergic inflammation.

## Bidirectional modulation between cysteinyl leukotrienes and other inflammatory mediators

**Fig. 2:**
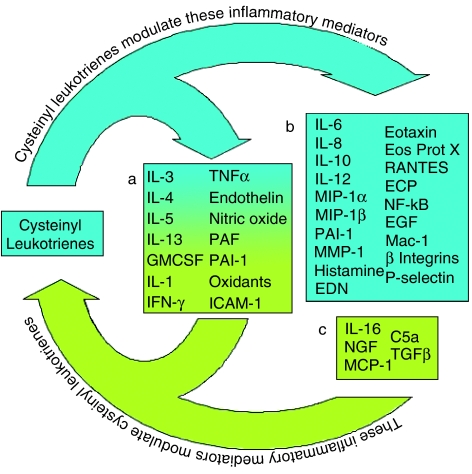
Interactions between cysteinyl leukotrienes (CysLTs) and inflammatory mediators. (a) Studies have demonstrated bidirectional regulation of these mediators; i.e., activity of these mediators can be modulated by CysLTs and, in turn, these mediators can modulate CysLTs activity. (b) Studies have demonstrated that the activity of these mediators can be modulated by CysLTs. (c) Studies have demonstrated that these mediators can modulate activity of CysLTs.

A complex network of interactions exists between CysLTs and a variety of inflammatory mediators (Fig. 2).

### Cysteinyl leukotrienes enhance the production and activity of inflammatory mediators

In patients with established allergic inflammation, immune responses to allergens are TH_2_ polarized, resulting in a preponderance of TH_2_ relative to the TH_1_ cytokines [122, 123]. *In vitro* and *in vivo* evidence suggests that TH_2_ cytokines can be modulated by CysLTs. *In vitro*, CysLTs or CysLT_1_ receptor antagonism have been shown to modulate the production of IL-3 [124], IL-4 [124, 125], IL-5, [124, 126], IL-10 [127], and GM-CSF [113, 124]. In patients with perennial AR, 4 weeks of treatment with pranlukast suppressed nasal mucosal production of IL-4 and IL-5 [46]. A 2-week treatment with montelukast decreased IL-4 and IL-13 levels in nasal lavage secretions from children with AR [120]. Also, serum IL-5 levels were reduced in children with asthma after 6 weeks of treatment with montelukast [129].

CysLTs may also affect a variety of non-TH_2_ mediators that play a role in inflammation associated with AR. The levels of the pro-inflammatory cytokine IL-6 were decreased from peripheral blood mononuclear cells [130] and those of the TH_1_ cytokine IFN-γ were increased from mononuclear cells [131] from healthy volunteers by CysLT_1_ antagonism with pranlukast and montelukast, respectively. In patients with AR, treatment with pranlukast suppressed production of IL-1β and IL-8 in the nasal mucosa [46], and treatment with montelukast increased IFN-γ levels in nasal secretions [128]. The increased production of IFN-γ in 5-LO knockout mice supports the regulation of this cytokine by products of the 5-LO pathway [132]. The level and activity of a variety of other mediators have been shown to be modulated by CysLTs. For example, several *in vitro* studies have demonstrated that levels of TNF-α produced by mast cells [126] and macrophages [48] are enhanced by CysLTs and decreased by CysLT_1_ receptor antagonism [48, 126, 133]. In patients with perennial AR, 4 weeks of treatment with pranlukast suppressed nasal mucosal production of TNF-α [46]. NF-κB is a transcription factor involved in regulating expression of proinflammatory cytokines such as IL-1, IL-6, IL-8, and TNF-α. Pranlukast and MK-571 have been shown to inhibit NF-κB activation in monocytes [130, 133]. In human mast cells, LTC_4_ and LTD_4_ increased the release of macrophage inflammatory protein-1β (MIP-1β), and this was blocked by MK-571 [126]. RANTES, which is produced by T cells, is a potent chemoattractant for monocytes, lymphocytes, and eosinophils. The level of RANTES in nasal mucosa of patients with perennial AR was decreased after 4 weeks treatment with pranlukast [46].

CysLTs have also been shown to affect mediators of inflammatory tissue growth and repair. For example, the proliferative effects of epidermal growth factor (EGF) on smooth muscle cells in culture were potentiated by LTD_4_ [134]. The proliferative effects of insulin-like growth factor (IGF) on smooth muscle cells in culture were also potentiated by LTD_4_ induction of matrix metalloproteinases (MMP-1) [135]. Insulin-like growth factor binding proteins (IGFBP) limit the ability of IGF to enhance differentiation, growth, and proliferation of cells. Proteolysis of IGFBP by MMP-1 removes inhibition of the IGF effects. Plasminogen activator inhibitor type-1 (PAI-1) promotion of abnormal tissue repair plays a role in airway remodeling; LTD_4_ increased, and montelukast decreased, production of PAI-1 by mast cells [136].

There is evidence for an interaction between CysLTs and histamine, another pivotal mediator of allergic reactions. LTD_4_ enhanced histamine-induced elevation of cytosolic calcium levels in cultured embryonic carcinoma cells [137] and prostaglandin E_2_ (PGE_2_) production from human monocytes and smooth muscle cells, as well as mouse macrophages [138]. The LTD_4_-enhanced histamine-induced PGE_2_ production was coincident with the appearance of additional histamine receptors [138]. These *in vitro* observations are in concordance with the *in vivo* effects of CysLT_1_ antagonism on nasal responsiveness to histamine described earlier [67]. The modulation of endothelin by CysLTs has also been demonstrated [139].

Exhaled nitric oxide (NO) is a marker of airway inflammation. Montelukast has been shown to reduce levels of exhaled NO in clinical trials with asthmatic adults [140, 141] and children [142, 143], but no studies have evaluated whether nasal NO is also affected. *In vitro*, LTC_4_ increased NO release from polymorphonuclear leucocytes [144] and from macrophages [145]. Ethacrynic acid, an inhibitor of LTC_4_ production, has been shown to inhibit NO production by mouse peritoneal macrophages [146]. Ovalbumin (OVA) challenge in OVA-sensitized rats increased lung-inducible nitric oxide synthase (iNOS) expression, which was decreased by treatment with montelukast [147]. Taken together, these data suggest a mechanism for the reduction in eNO observed clinically with montelukast. Superoxide radical levels in eosinophils have also been shown to be increased by LTD_4_ [100] and blocked by pranlukast [120].

### Inflammatory mediators enhance the production and activity of cysteinyl leukotrienes

In addition to the effects of CysLTs on other inflammatory mediators, the converse is also true, in that various inflammatory mediators can exert regulatory effects on CysLTs. Several studies have demonstrated the ability of TH_2_ cytokines to enhance the synthesis of CysLTs as well as the expression of the CysLT_1_ receptor. IL-5 increases the expression of FLAP and the translocation of 5-LO to the nucleus of eosinophils, which is accompanied by an increase in CysLT synthesis [148]. IL-3, IL-4, and IL-5 augment CysLT production by mast cells through induction of LTC_4_ synthase and 5-LO nuclear translocation [57]. The combination of IL-3 and C5a stimulated the production of LTC_4_ in basophils [149]. IL-3 [13, 149, 150], IL-5 [13, 149], and GM-CSF [149, 151, 152] stimulated CysLT synthesis in eosinophils, basophils, and T lymphocytes. GM-CSF also stimulated LTC_4_ synthesis through increased PLA_2_ mobilization of arachidonic acid in macrophages [153] and increased CysLT synthetic capacity through increased 5-LO [154] and FLAP expression [154, 155] in monocytes and neutrophils. TH_2_ cytokines also up-regulate CysLT_1_ receptors, a mechanism that, theoretically, can enhance CysLT actions. IL-5 [156], IL-4 [21, 126], and IL-13 [21, 157] up-regulated the expression of functionally active CysLT_1_ receptors on HL-60 cells differentiated into eosinophils (IL-5), monocytes (IL-4 and IL-13), macrophages (IL-4 and IL-13), and smooth muscle cells (IL-13). In support of the interaction between IL-13 and CysLTs, leukotriene receptor antagonism with MK-571 inhibited IL-13-induced CysLT synthesis in bronchoalveolar lavage (BAL) fluid in a mouse model of asthma [158]. The full range of interaction between TH_2_ cytokines and leukotrienes was illustrated in an *in vitro* study, which demonstrated that IL-13 increased CysLT_1_ receptor expression on lung-derived fibroblasts, subsequently enabling the cells to respond to LTC_4_ stimulation by releasing functionally active eotaxin, which subsequently promoted eosinophil chemotaxis and migration [111]. However, CysLT_1_ receptors have not been observed on nasal polyp-derived fibroblasts [159].

Non-TH_2_ inflammatory mediators also regulate CysLT synthesis and receptor activity. CysLT_1_ receptor expression on smooth muscle cells and endothelial cells has been demonstrated to increase when stimulated with IFN-γ [157, 160] and IL-1β [161]. IL-16 is increased in nasal mucosa of patients with AR during seasonal allergy exposure [162] and is a chemoattractant for eosinophils. In human eosinophils, IL-16-stimulated eotaxin release was followed by activation of CCR3 receptors and enhanced LTC_4_ and IL-4 release. These data suggest that IL-16-stimulated LTC_4_ and IL-4 release may occur through autocrine eotaxin activation of CCR3 receptors [163]. Transforming growth factor β1 (TGF-β_1_) and, to a lesser extent, TGF-β_2_ up-regulated 5-LO activity in HL-60 cells induced to granulocytic differentiation by dimethyl sulfoxide [164], LTC_4_ synthase expression in THP-1 macrophages [165], and CysLT_1_ receptor expression in smooth muscle cells [157]. The ability of TGF-β_1_ and LTD_4_ to synergistically enhance smooth muscle proliferation [157] functionally illustrates the inter-regulation of these two mediators. TNF-α [166], MCP-1 [149], C5a [149], platelet-activating factor (PAF) [167, 168], and endothelin [169] have been shown to enhance CysLT production by eosinophils, basophils, and mast cells, whereas nerve growth factor (NGF) [166] and oxidants [170] have been shown to reduce CysLT production. Finally, NO has been shown to increase CysLT production from human mast cells [171].

## Summary/conclusion

A substantial body of research reviewed in this article indicates that CysLTs satisfy Koch's postulates as mediators of AR, as (i) they are overproduced in the nasal mucosa of patients with the disease; (ii) they reproduce many clinical features of AR; and (iii) pharmacologic agents that block their synthesis or receptor-mediated actions attenuate the manifestations of AR. Recent studies have also elucidated a variety of mechanisms, other than direct symptom production, by which CysLTs promote AR. They have revealed that these lipid mediators participate in the genesis of systemic immune responses to antigen and in leucocyte accumulation, survival, and activation in affected tissues. One particularly compelling, but underappreciated, aspect of the involvement of CysLTs in allergic disease is the bidirectional interplay between CysLTs and other inflammatory mediators, such as cytokines, chemokines, growth factors, histamine, and reactive oxygen and nitrogen species. In this regard, leukotrienes can modulate the generation of a variety of mediators, and other mediators can modulate leukotriene actions by influencing both their synthesis and the expression of their receptors. Although a role for CysLTs in the pathogenesis of asthma was recognized first – involving many of these same mechanisms – the subsequent recognition of their role in AR supports the concept of a unified airway response to common triggering events.

It should be clearly stated that CysLTs represent only one of the participants of the allergic response. Other biologic products, including histamine or PGD_2_, play important roles. For example, histamine, acting through its H_1_ receptors, not only generates acute nasal symptoms, but it also has several properties that are not identifiable on the basis of its acute action on the nasal mucosa, including immunomodulatory activities and interactions with other mediators [172, 173]. CysLT_1_ receptor antagonists, like H_1_ receptor antagonists, have well-established clinical effects in AR. In fact, their overall clinical effectiveness appears to be of similar magnitude [174]. These antagonists are less effective compared with nasal glucocorticosteroids because the latter agents have a wider target spectrum. It should be kept in mind, however, that the systemic nature of treatment that CysLT_1_ receptor antagonists and antihistamines provide may have additional benefits that are not identifiable by the short-term studies that target the symptoms of AR [175]. This concept requires exploration.
